# Immune Complex Vaccine Strategies to Combat HIV-1 and Other Infectious Diseases

**DOI:** 10.3390/vaccines9020112

**Published:** 2021-02-02

**Authors:** Alex F. Tang, Gospel Enyindah-Asonye, Catarina E. Hioe

**Affiliations:** 1 School of Medicine, University of California, San Francisco, 533 Parnassus Ave, San Francisco, CA 94143, USA; Alex.Tang@ucsf.edu; 2 Division of Infectious Diseases, Department of Medicine, Icahn School of Medicine at Mount Sinai, 1 Gustave L. Levy Place, New York, NY 10029, USA; gospel.enyindah-asonye@mssm.edu; 3 James J. Peters VA Medical Center, 130 West Kingsbridge Road, Bronx, NY 10468, USA

**Keywords:** immune complex, antibodies, Fab, Fc, HIV-1, vaccine, steric, allosteric

## Abstract

Immune complexes (ICs) made of antibody-bound antigens exhibit immunomodulatory activities exploitable in a vaccination strategy to optimize vaccine efficacy. The modulatory effects of ICs are typically attributed to the Fc fragments of the antibody components, which engage Fc receptors, complement and complement receptors on various immune cells. These Fc-mediated functions facilitate the critical interplay between innate and adaptive immune systems to impact the quality and quantity of the elicited adaptive responses. In addition to the Fc contribution, the Fab fragment also plays an immunoregulation role. The antigen-binding domains of the Fab fragment can bind their specific epitopes at high affinity to sterically occlude these antigenic sites from recognition by other antibodies. Moreover, the Fab-mediated binding has been demonstrated to induce allosteric alterations at nearby or distant antigenic sites. In this review article, we survey published studies to illuminate how the immunomodulatory functions of ICs have been investigated or utilized in a vaccination strategy to fight against an array of infectious pathogens, culminating with IC vaccine designs aimed at preventing HIV-1 infection. In particular, we highlight IC vaccine candidates that exploit Fab-mediated steric and allosteric effects to direct antibody responses away or toward the V1V2 domain, the V3 loop, and other antigenic sites on the HIV-1 envelope gp120 glycoprotein. Like other HIV-1 vaccine approaches, the path for IC-based vaccines to reach the clinic faces major hurdles yet to be overcome; however, investigations into this vaccine strategy have provided insights into the multifaceted activities of antibodies beyond their conventional roles in the host defense against HIV-1 and other microbial pathogens.

## 1. Fc- and Fab-Mediated Activities of Immune Complexes

**Figure 1:**
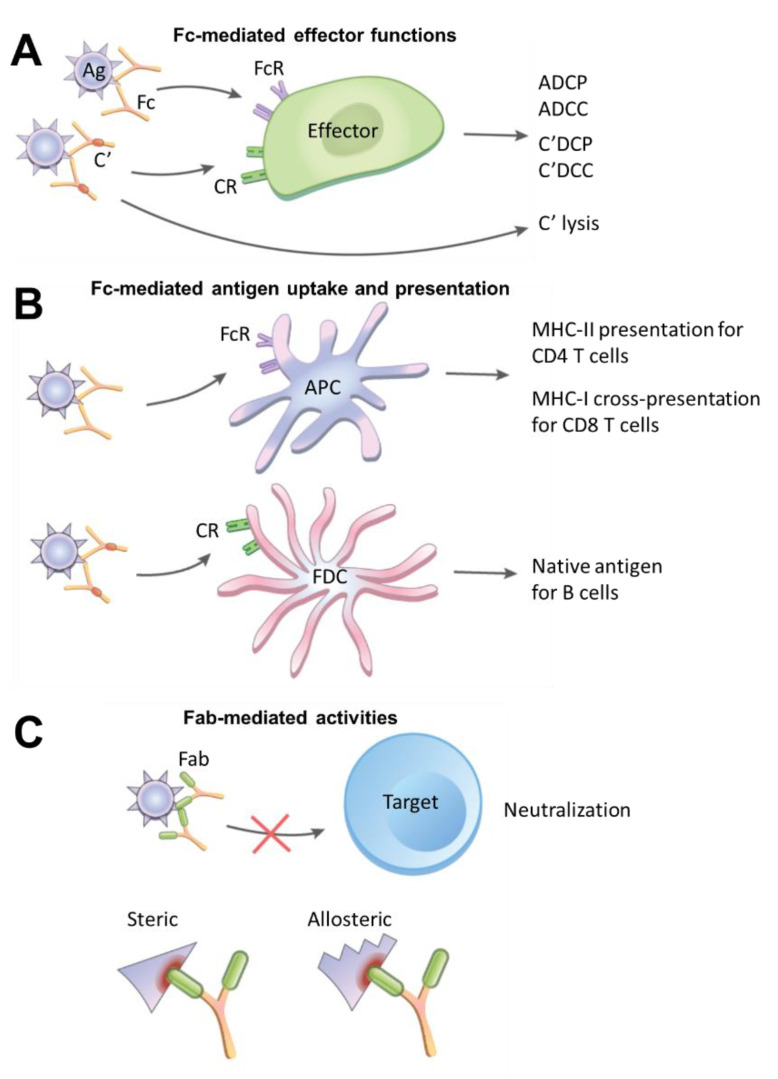
Fc- and Fab-mediated activities of immune complexes (ICs). (**A**) Fc-mediated effector functions for destruction of pathogens. ICs made of antibody-coated antigens (Ag) bind and cross-link Fc receptors (FcR) on effector cells such as macrophages, NK cells, and neutrophils. Interaction with FcgRIIa on macrophages, for example, can lead to IC ingestion by the antibody-dependent cellular phagocytosis (ADCP) mechanism. IC formed on virus-infected cells, on the other hand, may bind FcgRIIIa on NK cells to trigger NK cell degranulation and cause antibody-dependent cellular cytotoxicity (ADCC). In addition, ICs may activate the complement (C’) cascade to facilitate complement-dependent cellular phagocytosis (C’DCP) and cytotoxicity (C’DCC) by interacting with complement receptors (CR) on effector cells (e.g., CR3 on monocytes, CR4 on NK cells [1]) or to induce direct complement-mediated lysis. (**B**) Fc-mediated antigen uptake and presentation. ICs interact with FcRs, either type I (FcgRI, FcgRII, FcgRIII) or type II (DC-SIGN and CD23), on antigen-presenting cells (APCs) to promote antigen uptake. The APCs proteolytically process antigens for major histocompatibility complex (MHC)-II presentation to helper CD4 T cells and for MHC-I cross-presentation to cytotoxic CD8 T cells. Alternatively, ICs are opsonized with complement and captured by follicular dendritic cells (FDCs) via CR1 and CR2 on the FDC surface. The FDCs preserve native Ags in the form of ICs and present them to B cells. (**C**) Fab-mediated activities. Antibodies that bind pathogens and form ICs with their Fab fragments can neutralize the pathogens to prevent infection of target cells. Fab-mediated binding also imposes steric hindrance which alters the accessibility of specific antigenic epitopes on ICs. In addition, Fab-antigen interactions may induce allosteric alterations, resulting in increased or decreased structural stability, flexibility, or exposure of certain epitopes on ICs.

Immune complexes (IC) are formed naturally when antibodies are generated and bind to their specific antigens. An IC may be made of a single antibody targeting an antigen or many antibodies binding simultaneously to an antigen or antigens on the surface of a microbe. The presence of multiple antibodies on an immune complex promotes their avidity for Fc receptors and enables the cross-linking of these receptors on B cells, dendritic cells, follicular dendritic cells, natural killer (NK) cells, macrophages, neutrophils, or other types of immune cells. The Fc receptor engagement by ICs triggers a cascade of intracellular signals critical for Fc-mediated effector functions against microbes, such as antibody-dependent cellular phagocytosis (ADCP) and antibody-dependent cellular cytotoxicity (ADCC) (Figure 1A).

There are five major antibody isotypes, IgM, IgG, IgA, IgD, and IgE, which are classified based on their unique Fc fragments and each of which interacts with distinct Fc receptors (FcRs). IgG, the most abundant antibody in the blood, is further divided into four subtypes, IgG1, IgG2, IgG3, and IgG4, while IgA consists of two subtypes, IgA1 and IgA2. The Fc receptors are categorized into type I and type II, which bind the Fc domain at different interaction sites, conformation states, and stoichiometry [2]. The type I FcRs bind IgG in a 1:1 stoichiometric complex at its hinge-proximal CH2 region when the Fc domain adopts an open conformation [3]. Fc structural flexibility is controlled by the Fc-associated glycan and specifically the heterogeneity of the glycan oligosaccharide composition [4,5]. The type I FcRs for IgG include the canonical receptors such as FcγRI (CD64), FcγRIIA (CD32), FcγRIIB (CD32), FcγRIIIA (CD16a), and FcγRIIIB (CD16b), which differ in cell distribution and affinity for IgG subtypes. For example, FcγRI is a high-affinity receptor that binds mainly IgG1 and IgG3 and is expressed on dendritic cells, macrophages, neutrophils, and eosinophils [6]. On macrophages, the engagement of FcγRI and also FcγRIIA stimulates ADCP [6]. The low-affinity FcγRIIB, on the other hand, is expressed on B cells as a negative regulator of antibody production. FcγRIIIA on NK cells and some macrophages serves as the key receptor for inducing ADCC [7,8]. In contrast, the type II FcRs, which include C-type lectins DC-SIGN and CD23 (FceRII) [9], bind the Fc domain of IgG in a close conformation at the CH2–CH3 interface in a 2:1 stoichiometry. Expressed on myeloid dendritic cells, DC-SIGN facilitates the uptake of ICs. CD23, on the other hand, is expressed on B cells, T cells, follicular dendritic cells (FDCs), macrophages, NK cells, and a variety of other hematopoietic cells. CD23 also serves as an Fc receptor for IgE and captures IgE-bound antigens for phagocytosis, although on B cells, CD23 engagement may downregulate B cell activation and proliferation [10].

In addition to direct pathogen-killing actions described above, ICs serve as a bridge between innate and adaptive immune cells to boost the induction of the adaptive humoral and cellular immune responses against microbes. By engaging Fc receptors on antigen-presenting cells (APCs) such as dendritic cells and macrophages (Figure 1B), ICs facilitate the uptake of antigens and influence the efficiency of antigen processing for MHC-II presentation to helper CD4 T cells [11], which in turn supports the elicitation and development of CD8 T cells and B cells. The interaction with FcγRs on dendritic cells also induces dendritic cells’ maturation, as marked by enhanced expression of MHC-II and costimulatory molecules CD40, CD80, and CD86 [12], all of which play a critical role in the antigen-driven interaction between APCs and T cells. Likewise, FcγR-mediated uptake of ICs by macrophages has been shown to result in higher levels of antigen-specific CD4 T cell activation, as measured by IL-2 secretion, compared with the treatment of macrophages with antigen alone [13]. IC uptake by APCs also can lead to MHC-I cross-presentation for the activation of CD8 T cells. In this case, upon binding and crosslinking FcRs on the APC surface, ICs are internalized to the exogenous antigen processing pathway. Within a low pH environment, antigens detach from ICs and are shuttled to the cytosolic proteasomes for processing into peptides. The peptides then undergo transporter associated with antigen processing (TAP)-dependent transfer into the endoplasmic reticulum, where they are loaded onto MHC-I [12].

Working through the complement system, ICs are also integral to the host mechanisms of clearing or retaining microbes and microbial antigens. The binding of C1q to Fc fragments on ICs activates the downstream complement cascade resulting in the deposition of C3 and C4 degradation products, leading to the generation of C5 convertase and the assembly of the membrane-attack complex for initiating complement-mediated lysis (Figure 1A). Additionally, complement-opsonized ICs may interact with complement receptor 1 (CR1) and complement receptor 2 (CR2). ICs bound to CR1 on erythrocytes are carried to the spleen or liver for macrophages’ removal and degradation [14]. On leukocytes, CR engagement may trigger effector functions, such as complement-dependent cell-mediated phagocytosis (C’DCP) and complement-dependent cellular cytotoxicity (C’DCC) [15,16], which are complementary to FcR-mediated ADCP and ADCC activities. Indeed, Fc fragments engineered to bind complement but not FcRs have been shown to mediate antigen clearance at comparable kinetics and efficacy to antibodies with FcR-binding capacity [16].

Interestingly, on follicular dendritic cells (FDCs), C3-coated ICs are not cleared but accumulate, mainly through CR1, although CR2 and FcgRIIB also contribute to some degrees [17] (Figure 1B). FDCs are found in primary follicles and in germinal centers of secondary and tertiary lymphoid organs, including the spleen, lymph nodes, and other gut-associated lymphoid tissues, where they trap native antigens in the form of ICs to generate long-lasting antigen depots for B cells. Notably, ICs are captured initially by non-cognate B cells and delivered from the B cells to FDCs [18]. Mature B cells, especially marginal zone B cells and follicular B cells, primarily express CR2, which serves as a non-cognate receptor to capture ICs and subsequently shuttle them onto FDCs for prolonged antigen storage. During germinal center reaction, antigen-specific B cells access antigens on IC-bearing FDCs, and their B cell receptors undergo affinity maturation by somatic hypermutation. Operating as APCs, these B cells may further internalize and process antigens for MHC-II presentation to helper CD4 T cells [19,20].

While the immune functions of ICs are ascribed customarily to their Fc contribution, the Fab-mediated effects are important to consider as well (Figure 1C). Indeed, the key function of antibody is to bind and neutralize infectious microbes to prevent infection of the host cells, and this is mediated by antigen-specific Fab regions. In addition, Fab-induced steric and allosteric effects on antigen processing play a role in IC immunomodulatory activities. ICs formed with high-affinity antibodies, in particular, do not readily dissociate upon uptake into the endosomal compartment of APCs, and the presence of such antibodies has been shown in early studies from the 1980s to impact antigen processing. Using beta-galactosidase as a model antigen and 43 combinations of monoclonal antibodies and CD4 T cell hybridoma clones specific for distinct beta-galactosidase epitopes, Manca et al. observed that ICs potentiated antigen presentation and T cell stimulation in the majority of cases [13]. However, suppression was noted with some combinations of monoclonal antibodies and T cell clones, as well as in a subsequent experiment with polyclonal antibodies under an antibody-excess condition [13,21]. These studies provided initial evidence for the regulatory influence of ICs on antigen processing and presentation for T cells, which is mediated through the steric hindrance of proteolytic processing requisite for producing T cell peptide epitopes.

Since ICs retained on FDCs provide sampling of antigens to B cells, antibodies on ICs influence the accessibility of B cell epitopes on the bound antigens through steric or allosteric effects. Indeed, pre-existing antibodies from maternal–fetal transmission or through previous antigen exposures have been demonstrated to modulate the immunogenicity of live attenuated viral vaccines or viral vaccine vectors [22]. In addition to blunting infection of the viral vaccines, these antibodies form ICs to compete with or alter epitopes recognized by de novo antibodies and B cells, while also stimulating the activating or inhibitory Fc receptors on APCs and B cells. These mechanisms can impact the newly induced antibody responses in terms of potency, fine specificities, affinity, and effector functions. Further investigations into IC immunomodulatory activities are thus imperative if we seek to discern selective features of ICs to harness or exclude in order to exploit ICs as a versatile strategy to improve the efficacy of vaccines and also as immuno-therapeutics against infectious diseases, cancers, and other diseases.

## 2. Immune Complex Vaccines against Human and Animal Pathogens

Given the capacity of ICs for enhancing antigen uptake, MHC presentation, and APC maturation, ICs have been investigated and developed as vaccine immunogens in order to improve the immunogenicity and efficacy of vaccines against several human and animal pathogens. To this end, these IC vaccines primarily take advantage of the Fc immunomodulatory functions, yielding varying degrees of success. Table 1 lists candidate IC vaccines that have been tested in human clinical trials or animal experiments against non-HIV pathogens in publications from 2000 to 2020.

Among human vaccines, the IC vaccine that has advanced to human clinical trials is the hepatitis B virus (HBV) vaccine to treat chronically infected patients. Since HBV viral antigens do not elicit effective immune responses capable of clearing the virus in HBV-infected patients, it was postulated that immunization with viral antigens complexed with antibodies would lead to enhanced antigen uptake by APCs via the Fc receptor to result in improved antigen presentation and more effective induction of T cell responses [23]. To this end, Wen et al. generated a therapeutic immune complex vaccine (YIC) using yeast-derived hepatitis B surface antigens (HBsAg) and high-affinity antibodies derived from HBsAg hyper-immunized donors. In their Phase I trial with healthy adults, administration of up to six doses of 90 µg YIC generated serum anti-HBsAg antibodies in all recipients and increased serum levels of interferon (IFN)-gamma and IL-2, with no effect on blood chemistry or renal and liver functions [24].

In the subsequent Phase IIa trial with HBeAg-positive hepatitis B patients, five of ten participants who received the YIC experienced a marked decrease in viral load and development of antibodies against HBeAg, two of whom also developed antibodies against HBsAg and four of whom showed Th1/Th2 cytokine secretion. Four of the five responders also developed alanine aminotransferase (ALT) flares between 4 and 12 weeks after immunization, which the authors attributed to vaccine-induced cytolytic response, although two of the ten participants who received the placebo also developed ALT flares. In addition, incubation of patient peripheral blood mononuclear cell (PBMC)-derived dendritic cells with YIC resulted in the expression of functional and maturation markers (HLA-II, CD80, CD86, CD40, CD83), indicating the capacity of YIC to stimulate dendritic cell maturation and enhance their antigen presentation potential [25]. A phase IIb trial was conducted based on these data, with 65 HBsAg- and HBeAg-positive chronic hepatitis B patients receiving six injections of the YIC. Loss of HBeAg, presence of anti-HBe antibody, or suppression of HBV DNA were used as primary endpoints, and both HBeAg seroconversion (loss of HbeAg plus anti-Hbe antibody) and HBV DNA suppression as secondary endpoints. While there was no significant difference between the immunized and control groups in either primary or secondary endpoints, patients immunized with the highest dose (60 µg YIC) demonstrated a statistically greater HBeAg seroconversion rate compared to placebo (21.8% vs. 9%) [26].

To assess the therapeutic efficacy of the YIC vaccine and the potential of increasing the YIC dosage, a phase III trial was conducted, with 303 HBsAg- and HbeAg-positive patients receiving twelve injections, compared to six in the phase IIb trial. Only 14.0% of the immunized patients achieved HBeAg seroconversion, a marked decrease from the phase IIb trial results. Furthermore, YIC immunization decreased IL-17A expression in patients throughout the study, indicating the lowered therapeutic efficacy of twelve YIC doses, compared to six, attributed to immune fatigue [27]. This phase III trial demonstrated that merely increasing the dosage of YIC could not enhance its therapeutic efficacy and that further investigations would be needed to improve the YIC immunogenicity, especially in terms of induction of effective cell-mediated immune responses. Of note, neither the phase IIb nor the phase III trial assessed cytolytic T cell function or dendritic cell HBsAg presentation in immunized patients, which would have provided information about the key immune parameters postulated to contribute to the therapeutic efficacy of this IC vaccine strategy. A follow-up study to examine serum IgG Fc glycosylation in the phase III trial participants demonstrated that subjects who responded to YIC with HbeAg seroconversion had sustained increase of serum galactosylated IgG, which correlated with IL-2 upregulation, while non-responders did not, indicating the potential contribution of T cell responses to vaccine responsiveness [28]. Notably, Fc galactosylation of IgG has been shown to improve C1q binding and complement-mediated cytotoxicity and to enhance ADCC [29,30].

In the veterinary field, an IC vaccine (Bursaplex^®^) is commercially available for protecting chickens from infectious bursal disease virus (IBDV) infection [31]. This vaccine contains chicken embryo-derived live IBDV and IBD antiserum. A comparative study of Bursaplex against other IBD vaccines demonstrated that the administration of this IC vaccine subcutaneously to one-day-old chicks protected the animals from clinical symptoms and mortality after IBDV challenge. While control chicks who received no vaccine had a 20% mortality rate one week after IBDV challenge, and chicks who received live attenuated vaccine experienced a 10% mortality rate, chicks who received the IC had 0% mortality, similar to those receiving either turkey herpesvirus-vectored IBD vaccine (Vaxxitek^®^) or a combination of live attenuated and killed vaccines. Chicks immunized with either the IC or viral-vectored vaccine also had significantly better feed conversion ratios than non-immunized chicks and those receiving the live attenuated vaccine, with or without the killed vaccine. This occurred in spite of lower titers of antibody responses boosted after IBDV challenge in chicks that received IC or viral-vectored vaccines, which was attributed to the presence of interfering maternal antibodies. However, the viral-vectored vaccine was found to be superior to the IC and other tested vaccines in preventing bursal atrophy and bursal lesions.

A promising veterinary IC-based vaccine was also reported to protect pigs against porcine parvovirus (PPV) [32]. The IC vaccine was made of immunoprecipitated PPV virions bearing PPV virus protein 2 (VP2) and pig or rabbit anti-PPV polyclonal immune sera. Rabbits were included in the study for the initial immunogenicity evaluation. Rabbits immunized with the IC vaccine with rabbit Ig generated a robust immune response with anti-PPV antibody levels comparable to levels in rabbits that received the commercial inactivated vaccine (Parvokal^®^) at two weeks after vaccination and higher levels at three weeks. Rabbits that received the IC vaccine containing porcine IgG, on the other hand, produced a lower anti-PPV antibody level that was roughly half of those observed after immunization with the rabbit Ig-containing IC. When the vaccine with porcine IgG was administered intramuscularly to gilt pigs six weeks before mating, and two weeks later, anti-PPV antibody responses measured by hemagglutination inhibition (HI) assay were detected at levels comparable with those achieved with the commercial inactivated vaccine Parvokal^®^, although vaccine efficacy against virus challenge was not assessed. Of particular note, the IC vaccine contained half the inactivated vaccine’s viral content, indicating a dose-sparing advantage of the IC vaccine.

The IC strategy has also been investigated for vaccines against equine herpesvirus 1 (EHV-1) in a murine model [33]. The IC vaccines were constructed as a solid matrix-antibody-antigen complex consisting of a monoclonal antibody (mAb) bound via Fc to *Staphylococcus aureus*, and via Fab, to either EHV-1 glycoproteins C or D. Administration of both ICs to mice thrice intraperitoneally resulted in induction of antibodies against the respective glycoprotein with virus-neutralizing activity as well as antibodies that caused complement-mediated lysis of EHV-1-infected cells. Depending on the mouse strains and the EHV-1 glycoproteins used to form the IC vaccines, a skewing toward Th1, Th2, or Th1/Th2 response was observed as indicated by the prevalence of IgG2b or IgG1 responses. Splenocytes isolated from IC-immunized mice also proliferated in response to EHV-1, but proliferative responses declined for the gC group post-challenge as compared to the gD group. In addition, lymphocytes from cervical lymph nodes of the gC group, unlike those of the gD group, showed no proliferative response, implicating the involvement of suppressor T cells in response to the gC IC vaccine. Upon intranasal EHV-1 challenge, mice immunized with both IC vaccines showed fewer clinical signs of infection after viral challenge, compared to control. However, immunization with gD resulted in a roughly 30% reduction of virus load in turbinates, compared to control, while immunization with gC had no such effect. The reduction of clinical signs and virus titer in turbinates resulting from gD immunization was attributed to the high levels of neutralizing antibodies, local and systemic cell-mediated immune responses skewed toward a predominant Th2 response.

An IC vaccine strategy was likewise tested against the mucosal bacterial pathogen *Francisella tularensis* [34]. To produce the IC, inactivated *F. tularensis* (iFt) was complexed with mouse anti-*F. tularensis* LPS IgG2a mAb. Since FcγRs are expressed on mucosal APCs and FcRns to facilitate Ag delivery to mucosal lymphoid tissue, a mAb of IgG2a isotype capable of binding to all three FcγRs as well as to FcRn was used to form the IC vaccine. Compared to immunization with a non-complexed vaccine, immunization of mice with the IC resulted in a significantly greater survival rate, as well as reduced tissue inflammation and inflammatory cytokine production after bacterial challenge. Enhanced protection was dependent on Fc-FcR engagement, as removal of the IgG2a Fc domain eliminated the greater protective effect of the IC vaccine, yielding a level of protection comparable to that seen with the non-complexed vaccine. Moreover, mice lacking FcγRI/III or FcRn were not protected by immunization with the IC.

In addition, IC vaccines have been tested in mice for the capacity to elicit immune responses against tick-borne encephalitis (TBE) [35]. Three ICs were created, each with a different mAb against distinct epitopes on the soluble TBE envelope protein. There were no significant differences in the anti-envelope antibody titers of mice that received each of the IC vaccines compared to mice that only received the envelope protein. However, the fine specificities and cross-reactivity of elicited anti-envelope antibodies, as measured in an enzyme-linked immunosorbent assay (ELISA) against the TBE envelope protein (sE), envelope substructures DI, DIDII, and DIII, as well as whole TBE virion and heterologous envelope protein from West Nile Virus, differed in the groups of mice that received two of the IC vaccines, while no difference was observed for the third group. The notion of epitope-shielding or masking was postulated for the sE-B4 IC, in which the mAb B4 hid a particularly immunogenic epitope on the DIII lateral ridge, shifting the induction of antibody response to other epitopes. In support of this idea, an ELISA was conducted to test serum antibodies against wild-type DIII and against mutant DIII lacking the B4 binding site in the lateral ridge, which showed that sera from the sE-B4 IC group had a lower proportion of DIII lateral ridge antibodies compared to sera from the un-complexed sE group. Another IC formation (sE-A3) caused allosteric conformational changes, shifting the sE conformation and yielding an antibody response against the new conformation. As a result, immunization with sE-A3 yielded a lower antibody titer against free TBE virion, compared with immunization with sE alone. The study suggested that, beyond the Fc-mediated functions of ICs, the Fab-induced epitope-shielding and conformational changes also play a role, and the Fab-dependent effects may be more prominent when IC vaccines are constructed with mAbs rather than polyclonal antibodies used in the other IC vaccines described above [25,30,31,35]. IC vaccine strategies utilizing mAbs are discussed in depth below in the context of HIV-1 envelope vaccines.

**Table 1:** Immune complex (IC) vaccines against pathogens other than HIV-1.

Pathogens	Immune Complex Components	Dosing Regimen and Study Design	Conferred Effects	Additional Notes	References
Hepatitis B Virus (HBV)	Yeast-derived hepatitis B surface antigen (HBsAg) complexed to human anti-HBs immunoglobulin (HBIG)	Phase IIb trial; 6 IM injections of 30 µg, 60 µg, or placebo at 4-week intervals, follow-up for 24 weeks after the last injection	No significant difference of group effects between 60 µg group vs. placebo in 1° endpoints (loss of HBeAg or induction of anti-HBe antibody or suppression of HBV DNA) A significant difference in HBe seroconversion (loss of HBeAg and anti-HBe seropositive) between 60 µg group (21.8%) vs. placebo (9%) at end of follow-up	Follow-up phase III trial utilizing 12 IM injections of 60 µg IC yielded lower HBe seroconversion compared to 6 IM injections in phase IIb (14.0% vs. 21.8%)	[27,36]
Infectious Bursal Disease Virus (IBDV)	Bursaplex^®^ vaccine containing a live strain of IBDV of chicken embryo origin and IBD antiserum	1-day old commercial broiler chicks immunized SC with IC vaccine; compared with recombinant turkey herpesvirus vector vaccine (HVT-IBD, Vaxxitek^®^), live attenuated vaccine, and killed vaccine; challenged with IBDV at week 4	Bursaplex IC vaccine protected 100% of animals from clinical symptoms and mortality after IBDV challenge; still resulted in bursal atrophy and bursal lesions	IC vaccine resulted in low anti-IBDV antibody titers; viral vaccine component neutralized by maternal antibodies Vector HVT-IBD vaccine deemed superior, rendered 100% protection while avoiding bursal atrophy and lesions	[32]
Equine Herpesvirus-1 (EHV-1)	Solid matrix-antibody-antigen complex, consisting of MAb bound via Fc to *Staphylococcus aureus* and via Fab to EHV-1 glycoproteins C or D	BALB/c or C3H mice immunized IP on days 0, 28, and 43, followed by IN EHV-1 challenge on day 53	Both neutralizing and lysis-mediating antibodies elicited against respective glycoproteins, along with priming of EHV-1-specific local and systemic T cell proliferative responses 35% reduction of virus load in turbinates of mice immunized with glycoprotein D, but not of mice immunized with glycoprotein C	Formation of CD8+ T suppressor cells in the cervical lymph nodes of glycoprotein C-immunized mice In the lungs, no significant difference in virus load of mice immunized with glycoproteins D and C compared to control	[34]
Porcine Parvovirus (PPV)	Immuno-precipitate of <1000 kDa PPV viral particles and pig or rabbit anti-PPV polyclonal immune sera. IC consisted mostly of PPV VP2 protein and IgG	Gilts immunized IM six weeks before mating and boosted two weeks later; chinchilla rabbits immunized IM once	Generation of anti-PPV antibodies in gilts and rabbits starting from 2 weeks post-vaccination. IC-induced antibody titers comparable to titers induced by Parvokal^®^, a commercial inactivated PPV vaccine	IC vaccine was dose-sparing, using half the amount of virus in Parvokal	[33]
Tick-Borne Encephalitis Virus (TBEV)	TBEV sE protein complexed with IgG against one of three distinct epitopes	C57BL/6 mice immunized IP twice with an interval of 14 days, boosted 8 weeks later	IC-immunized mice generated comparable titers of serum anti-sE antibodies as mice immunized with sE only at 22 weeks post-immunization.	For two of the three IC vaccines, fine specificities of antibody responses differed from the sE-only vaccinated group. Evidence of antibody-induced conformational changes and epitope shielding in the two IC vaccines	[37]
*Francisella tularensis*	Inactivated bacteria complexed with mouse IgG2a anti-*F. tularensis* mAb	Mice immunized IN and boosted on day 21, followed by IN bacterial challenge on day 35	Significant enhancement in the survival of mice vaccinated with IC vaccine vs. non-complexed bacteria IC vaccination led to a 10-fold decrease in bacterial burden, tissue inflammation, and cytokine production; enhanced production of protective mucosal IgA	Immunomodulatory benefits of IC vaccine attributed to FcR targeting on mucosal antigen-presenting cells	[35]

IM: intramuscular, SC: subcutaneous, IP: intraperitoneal, IN: intranasal.

## 3. Immune Complex Vaccine Strategies against HIV-1

Since the start of the HIV-1 epidemic almost 40 years ago, the virus has infected an estimated 56 to 100 million people worldwide; furthermore, 25 to 42 million people have died from the disease caused by the virus, acquired immune deficiency syndrome (AIDS) [36]. One to two million individuals have been newly infected each year in the past decade, and more than 30 million people are now living with HIV-1 [36]. Efficacious vaccines needed to prevent infection and to control this global pandemic are not yet available. Vaccine-induced immune parameters critical to prevent or control HIV-1 infection also are not fully understood, although elicitation of antibodies against the virus envelope glycoproteins (Env) is one important component generally accepted to be required. Ideally, HIV-1 vaccines would induce durable antibody responses against multiple Env epitopes, and these antibodies would have multiple functions effective against a broad range of circulating isolates.

Efforts to develop HIV-1 vaccines have yielded unsatisfactory results. The most promising was the Thai RV144 trial, which yielded a vaccine efficacy of 60.5% for the first year and 31.2% at 3.5 years [38]. In this trial, healthy volunteers were administered four injections of a recombinant canary pox vector vaccine (ALVAC-HIV vP1521) and two injections of recombinant bivalent AIDSVAX clades B and E gp120 glycoproteins. This trial correlated reduced risk of heterosexual HIV-1 acquisition with high IgG levels against the V1V2 region of HIV Env gp120 [39]. However, the antibody levels declined soon after vaccination, corresponding to the observed loss of protection over time [39]. Induction of IgG antibodies against the gp120 V3 region also correlated with protection, albeit only in vaccinees with low levels of Env-specific plasma IgA and neutralizing antibodies. Notably, the RV144 vaccine did not induce broadly neutralizing antibodies, although non-neutralizing antibodies with Fc functions were detected [40,41], hinting at the potential contribution of Fc-mediated antibody activities to protection. Importantly, these results provide the first evidence that, like vaccines against many other pathogens, antibodies are a key immune correlate of vaccine efficacy against HIV-1 infection in humans. The findings also offer a defined pathway for improvement with the V1V2 and V3 regions of HIV-1 Env gp120 as important targets for the vaccine-induced antibody responses.

### 3.1. IC Vaccines to Elicit Antibody Responses against V3

A number of laboratories have explored the potential of the IC vaccine approach for optimizing the targeting of vaccine-induced antibodies to the desired Env epitopes. Table 2 summaries Env-based IC vaccines that have been tested in animal models to direct antibody responses toward or away from specific Env epitopes. In our initial experiments, we demonstrated the utility of specific anti-gp120 mAbs, when administered with clade B gp120 proteins as IC vaccines in mice, to increase or decrease the induction of antibody responses against V3 [42,43,44,45,46,47,48]. We showed that immunization with gp120 complexed with an anti-CD4-binding site (CD4bs) mAb resulted in faster induction and higher levels of antibodies against V3 as compared to gp120 alone [48]. The elicited antibodies had neutralizing activity against tier 1 viruses sensitive to anti-V3 Abs. Induction of antibody responses against other sites such as gp120 core lacking V1V2 and V3, V1V2, N-terminal C1, and C-terminal C5 regions was not enhanced [48]. Enhanced V3-specific antibody responses were consistently generated with IC vaccines made with each of the two anti-CD4bs mAbs tested (654D and 559/64D) and with two different gp120 strains (LAI and JRFL, both clade B). However, the V3-specific antibodies elicited by the JRFL IC vaccines were more cross-reactive and more capable of neutralizing heterologous tier 1 viruses than the antibodies produced in response to the LAI IC vaccine, which recognized only the autologous LAI virus [42,44,47]. Moreover, the IC-induced antibodies displayed higher avidity for gp120, as shown by greater resistance to a chaotropic agent, sodium thiocyanate [45]. An IC vaccine made of gp120 and V1V2-specific mAb (2158) also stimulated an augmented V3 antibody response despite a lower titer and affinity than the gp120/CD4bs mAb ICs, whereas the IC vaccine formed with an anti-C2 mAb (1006-30) did not cause any enhancement. In stark contrast, the IC vaccine formed with an anti-V3 mAb (694/98D or 1006-15D) blocked the induction of antibodies against V3 [42,48]. These studies demonstrated the importance of the mAb specificity as dictated by the Fab domain in determining the immunogenicity of these IC vaccines.

The contribution of the Fab domain was verified by testing IC vaccines made with F(ab’)_2_ fragment versus intact IgG of the anti-CD4bs mAb 654D [45]. Enhanced V3-specific binding and neutralizing antibody responses were induced to comparable levels, supporting the idea that the Fab component mediates this IC vaccine’s immunomodulatory activity. To further understand the mechanism by which ICs modulate immunogenicity of V3, we compared the V3 antigenicity on ICs made with mAbs of different specificities by probing with a mAb specific for the V3 crown, a highly immunogenic region distal from the V3 glycan epitopes targeted by broadly neutralizing antibodies such as PGT121-123, PGT125-128, and PGT130-131 [49,50,51]. The data revealed a significant increase in V3 recognition on gp120 complexed with each of the four CD4bs mAbs tested. Higher V3 antigenicity was also seen on ICs made with anti-V2 mAbs but not with anti-C2 mAbs, while unsurprisingly, V3 was blocked on ICs made with anti-V3 mAbs [42,48]. The altered V3 antigenic profiles were observed in ELISA and confirmed by biolayer interferometry, which provided more quantitative affinity measurements [42].

We also studied the effect of IC formation with different anti-gp120 mAbs on gp120 resistance to proteolytic degradation [44,52]. ICs made with anti-CD4bs mAbs displayed varying degrees of resistance to lysosomal enzymes, which corresponded with Fab affinity and IC stability. Notably, the gp120/CDbs 654D complex was one of the ICs with the highest affinity, greatest stability in an acidic environment, and highest resistance to proteases [52]. The increased resistance of this IC was further confirmed upon treatment with individual proteases abundant in lysosomes, such as cathepsin L, S, or D [44]. Altogether, the in vitro antigenicity and stability data imply that Fab-dependent mAb binding to distinct gp120 sites exerts allosteric and steric effects on V3, resulting in the modulation of V3 immunogenicity upon IC vaccine administration.

The role of antibody response to V3 in protection against HIV-1 infection remains controversial. The immunodominant V3 crown contains conserved elements but is often occluded on native Env trimers on the majority of HIV-1 isolates [53,54,55]. On the other hand, the V3 crown is accessible on most Env vaccines, including recombinant gp120 proteins, leading to prominent induction of anti-V3 crown antibodies in vaccinees [56]. The excessive immunodominance of V3 may hamper the host from mounting responses to the subdominant, more desirable, epitopes. Indeed, immunization of rhesus macaques with a combination of V1V2- and V3-scaffolded immunogens was found to induce lower antibody responses to V1V2 as compared to that with V1V2-scaffold immunogens alone [57]. Our studies described the effectiveness of IC vaccines made with clade B gp120 proteins and anti-V3 mAbs to suppress the induction of antibody responses to V3 [42]. However, the responses to V1V2, which was targeted by antibodies that correlated with a lower risk of HIV-1 infection in the RV144 vaccine trial, were not improved.

### 3.2. IC Vaccines to Elicit Antibody Responses against V1V2

**Figure 2:**
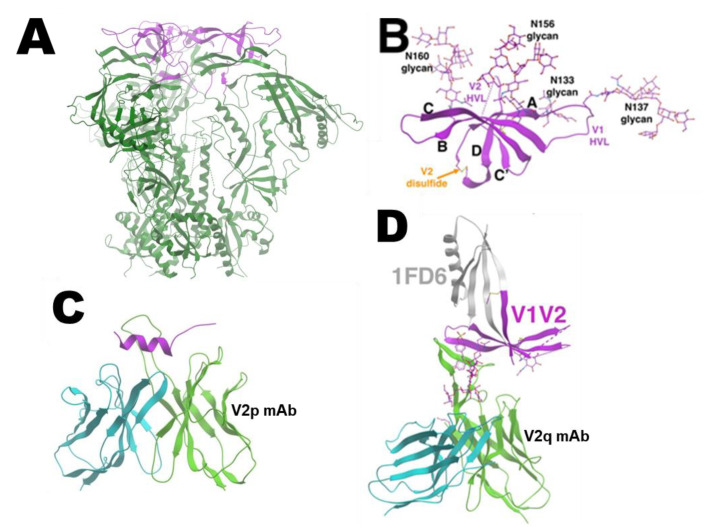
V1V2 structure. (**A**) Three V1V2 domains create the apex of HIV-1 Env. PDB: 6VY2 [64]. (**B**) Each V1V2 is a 5-strand β-barrel. PDB: 4YWG [59]. (**C**) The V2 C-strand is α-helical, when bound by V2p mAb. PDB: 4HPO [62]. (**D**) The binding of V1V2 apex or V2q mAbs like PG9 requires that the C-strand assume a β-sheet conformation. PDB: 3U36 [59]. Purple: V1V2, dark green: gp120 and gp41 subunits beyond V1V2, green and cyan: heavy and light chains of mAbs, gray: 1FD6 scaffold.

**Figure 3:**
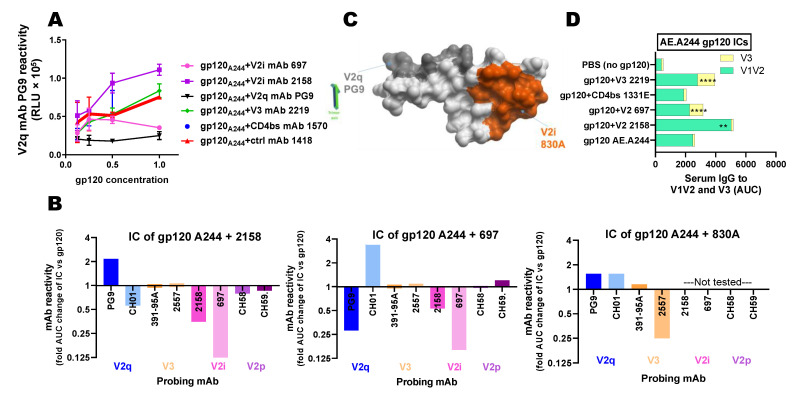
Altered immunogenicity and antigenicity of gp120 A244 upon IC formation. (**A**) Changes in PG9 reactivity of gp120 AE.A244 upon IC formation with distinct mAbs [42]. (**B**) Fold changes in mAb reactivity to gp120 AE.244 in complex with V2i mAbs 2158, 697, and 830A vs. un-complexed gp120 treated with control mAb 1418 as probed by V2q, V3, V2i, and V2p mAbs [42]. (**C**) Location of PG9 epitope at the top of the V1V2 apex near the trimer axis vs. V2i 830A epitope in the underbelly of V1V2 toward its distal end [59,60]. (**D**) Mice were immunized with gp120 AE.A244 alone or in complex with mAb (s.c. 4x) [42]. Sera from 2 weeks after the last injection were diluted and tested in ELISA for reactivity with V1V2 (V1V2 ZM109, Clade C) or V3 (A244, CRF_01.AE). Areas under curves (AUCs) were calculated from titration curves. **, *p* < 0.001; ****, *p* < 0.0001 vs. the gp120 group.

The V1V2 domain has a number of sites that elicit antibody responses in HIV-1-infected subjects and seronegative recipients of HIV-1 vaccine candidates. Three V1V2 domains, each of which is a modular 5-strand β-barrel on an Env protomer, create the apical cap of the trimeric Env (Figure 2A,B) [58,59,60,61]. One antigenic site of interest is the V2C strand capable of adopting polymorphic structures. This region is recognized by the V2p class antibodies that can recognize V2 peptides, such as mAbs CH58, CH59, and the CAP228 series, in α-helical configurations (Figure 2C) [62,63]. In contrast, the V2q class Abs, like mAbs PG9, CH01, PGT145, and PGDM1400, that preferentially bind to quaternary epitopes on the V1V2 apex of the Env trimer, require that the C-strand assume a β-sheet conformation (Figure 2D) [58,60,63]. The third known class is called V2i mAbs based on the recognition of epitopes near the integrin α4β7-binding motif at the distal end of V1V2 [60]. In our recent studies, we observed that, contrary to clade B ICs, ICs made with gp120 A244, a clade E AIDVAX component of the RV144 vaccine, exhibited antigenic and allosteric changes, impacting V1V2 [42]. ICs of interest were comprised of gp120 A244 and V2i mAbs. ELISA results showed that mAb 2158 binding to gp120 A244 increased the reactivity of V2q mAb PG9, while the IC of gp120 A244 and another V2i mAb 697 lowered PG9 reactivity (Figure 3A) [42]. ICs formed with V3- or CD4bs-specific antibodies did not show such alterations. Comparison of ICs made with gp120 A244, and three different V2i mAbs (Figure 3B), showed further evidence of the mAb-specific allosteric changes. The gp120 A244 and 2158 IC had higher PG9 recognition and lower reactivity of another V2q mAb, CH01, without altering reactivity of V3 or V2p mAbs. The opposite pattern was observed with IC of gp120 A244 and 697, which lowered PG9 binding and augmented CH01 reactivity. A third V2i mAb, 830A, induced yet a different effect, modestly enhancing the binding of both PG9 and CH01 while reducing the reactivity of V3-specific 2557. The crystallographic data available for V2i mAb 830A show that the V2i mAb recognizes V1V2 at a distinct site and with a different angle of approach as compared to PG9 (Figure 3C) [60], supporting the notion that V2i mAb binding to gp120 A244 exerts allosteric changes on the V1V2 structure, which promotes or impedes the V2C β-sheet conformation required for the binding of V2q mAbs such as PG9 and CH01.

Immunization of mice further demonstrated immunogenic alterations on V1V2 upon IC formation of gp120 A244 and different mAbs. Notably, gp120 A244 complexed with V2i mAb 2158 elicited the highest levels of V1V2-specific serum antibody responses that were cross-reactive with clades B, C, and E (Figure 3D) [42]. The enhanced antibody response observed against overlapping V2 peptides was mapped to the C-strand in the V1V2 domain, which mainly adopts the a-helical conformation, i.e., the V2p-type epitopes [42]. The elicited antibodies also recognized V1V2 on the 1FD6 scaffold that presents the V2C strand in the beta-sheet conformation but did not compete with V2q mAb PG9, indicating the presence of V2i-type Abs. The total IgG responses to gp120 were unchanged [42]. ICs made with gp120 A244 and V2i mAb 697 or CD4bs mAb 1331E elicited V1V2-specific antibodies with lower cross-reactivity and more robust strain-specific V3 antibody responses than un-complexed gp120 A244 or the gp120 A244/2158 IC (Figure 3D). Sera from mice immunized with gp120 A244 ICs or gp120 alone had no neutralizing activity against tier 1 or tier 2 viruses, indicating the absence of broadly neutralizing PG9-like Abs, but displayed Ab-dependent cellular phagocytosis (ADCP) [42]. Hence, improved PG9 antigenicity seen on the gp120 A244/2158 IC was not sufficient to induce PG9-like antibody responses in vivo, although this IC improved the elicitation of V2i- and V2p-type antibodies capable of mediating Fc-dependent functions.

### 3.3. IC Vaccines to Elicit bNAb Responses

Several other labs have also explored the IC vaccine strategies by exploiting the Fab specificity to direct the induction of antibody responses against broadly neutralizing epitopes that are poorly immunogenic (Table 2). Chen et al. tested an IC vaccine consisting of a crosslinked gp120 core and human mAb 17b, a CD4i antibody specific for the bridging sheet, to induce bnAbs specific to the CD4 binding site (CD4bs), exemplified by the VRC01 mAb lineage [65]. Structural analyses reveal that the key difference between anti-CD4bs mAbs with neutralizing versus non-neutralizing activities is the angle of approach to access the CD4bs epitopes within the context of the native trimeric Env structure. The non-neutralizing anti-CD4bs antibodies approach and contact gp120 residues that are occluded in the native Env, including the bridging sheet. The 17b mAb was used to block the bridging sheet and preferentially expose the CD4bs for an altered angle of approach. Immunization of rabbits with the 17b-containing IC suppressed the induction of antibodies against the bridging sheet, elicited neutralization against tier 1 viruses, and also transiently induced an antibody response of a similar binding profile to VRC01-class CD4bs bnAbs [65]. However, the induction of broadly neutralizing VRC01-like antibodies remained unattained.

A similar study in guinea pigs tested IC vaccines made of cross-linked gp120 proteins of clade B BaL or 89.6 strains with mAb A32 against a CD4-induced epitope in the inner domain of gp120 [66], which stabilizes the CCR5 chemokine receptor binding site on gp120. While mAb A32 binding enhanced the exposure of the CCR5 binding site, titers of neutralizing antibodies were comparable in sera from the animals immunized with ICs and with gp120 alone [67]. Altogether, these studies demonstrate that stabilization of the CD4- or CCR5-binding sites can be triggered by binding certain mAbs to gp120. However, unlike those seen with V3 and V1V2 [42,43,44,45,47], the allosteric effects on CD4- and CCR5-binding sites did not lead to increased immunogenicity of these receptor-binding sites.

In the past decade, the use of native-like trimeric Env vaccines, rather than monomeric gp120 immunogens, has been advocated as a rational pathway to induce bNAbs, as these antibodies target epitopes present or accessible only on the functional trimeric Env spikes expressed on the virions. However, because the near-native Env immunogens such as the BG505 SOSIP.664 trimer display immunodominant strain-specific epitopes in a glycan-devoid region around positions 241 and 289 (“the glycan hole”), which elicit autologous neutralizing antibody responses, mAbs have been used to conceal this glycan hole in the constructed IC vaccines [68]. Immunization of rabbits with each of the two ICs made with glycan hole-specific rabbit mAbs generated lower levels of trimer-specific antibody responses with slower decay kinetics than immunization with un-complexed Env trimer, indicating suppression of antibody responses against the immunodominant glycan hole and diversion to other regions [68]. However, neutralizing activities were not increased in breadth or potency, signifying that the diverted answers did not extend toward cross-reactive neutralizing epitopes. Hence, similar to our efforts to suppress anti-V3 responses [42,48], the dominant responses against the glycan hole can be blocked using an IC approach, but the induction of antibody responses toward the more sought-after but less immunogenic Env regions requires additional strategies other than merely suppressing the dominant responses.

One strategy may be to construct IC vaccines with a combination of mAbs: mAbs imposing steric blockage against undesired dominant epitopes plus mAbs triggering allosteric alterations to augment the exposure and stability of the desired bNAb epitopes. Moreover, instead of aiming to induce solely bNAb-like responses, it would be more practical to utilize IC vaccines to stimulate cross-reactive multifunctional antibodies capable to mediate an array of antiviral activities that are characteristic of polyclonal vaccine-induced responses. For examples, the IC strategy may be employed to improve upon a number of trimeric Env vaccines reported to elicit neutralizing antibodies that are not as broad and potent as any of the bNAbs but were effective against some tier 2 heterologous viruses [69]. The approach may also be applied to vaccine candidates that stimulate non-neutralizing anti-Env antibodies with Fc functions such as ADCC, ADCP, and complement fixation [56,70,71].

**Table 2:** Immune complex (IC) vaccines against HIV-1.

Strategy	IC Components	Species Used in Study	Immunomodulatory Effects	Additional Notes	References
Targeting V3	gp120 JRFL or LAI (both clade B) + anti-CD4bs mAb 654	BALB/c mice	Both ICs elicited greater titers of antibody responses to V3 Anti-V3 antibodies generated by JRFL IC had limited neutralizing activity but were more cross-reactive than those by LAI IC	Enhanced V3 immunogenicity of ICs correlated with greater antigenicity	[44,49]
Targeting V3	gp120 JRFL (clade B) + anti-CD4bs mAb 654, anti-V2i mAb 2158, or anti-C2 mAb 1006-30	BALB/c mice	ICs with CD4bs mAb 654 or V2i mAb 2158 (to a lesser extent) enhanced V3 antibody responses IC with C2 mAb 1006-30 reduced V3 antibody responses	ICs with F(ab)_2_ 654 were sufficient in enhancing V3 antibody responses, highlighting the key role of Fab	[48]
Targeting V3 Masking V3 Targeting V1V2	gp120 JRFL (clade B) + anti-CD4bs mAb 654, anti-V2i mAb 2158 gp120 A244 (CRF_01.AE) + anti-V2i mAb 2158	BALB/c mice	JRFL ICs elicited V3 antibody responses of greater titers and breadth, and with more tier 1 virus neutralizing activity A244 ICs induced higher levels of V1V2 antibodies with some cross-reactivity	JRFL ICs with C2 or V3 mAbs reduced V3 antibody response JRFL and A244 ICs modulated antibody responses to V1V2 and V3 without affecting the overall antibody responses to HIV-1 Env	[43]
Stabilizing CD4i (chemokine receptor binding site)	gp120 BaL or 89.6 (clade B) + mAb A32	Outbred Harley guinea pigs	IC enhanced exposure and antigenicity of CD4i in vitro IC caused no change in CD4i immunogenicity and neutralizing antibody responses in vivo	Enhanced CD4i antigenicity in vitro did not translate to enhanced immunogenicity in vivo	[67]
Masking CD4i Targeting CD4bs bNAb (VRC01 lineage)	gp120 core + anti-CD4i mAb 17b	New Zealand White rabbits	IC suppressed antibodies against the CD4i bridging sheet, elicited tier 1 neutralization, transiently induced antibody response with similar binding profile to VRC01-class CD4bs bnAbs No enhanced and long-term induction of VRC01-like Abs	17b mAb blocked CD4i bridging sheet and non-neutralizing CD4bs while exposing CD4bs for VRC01 approach from an alternate angle	[70]
Masking glycan hole	BG505 SOSIP.664 gp140 trimer (clade A) + mAbs that target strain-specific glycan hole	New Zealand White rabbits	ICs elicited lower levels of strain-specific antibody responses, indicating successful blockage of immunodominant glycan-hole region ICs stimulated binding antibodies with a lower rate of decay	Diversion away from glycan hole did not improve antibody responses against cross-reactive neutralizing epitopes	[68]

### 3.4. Fc Functions in IC Vaccines

The IC vaccine studies described above demonstrate the prominent Fab-dependent steric and allosteric effects that modulate IC-induced antibody responses toward or away from HIV-1 Env regions of interest. Our own gp120 IC experiments provide clear evidence for the importance of the Fab fragment and specificity used to form the ICs in altering the antigenic and immunogenic properties of V1V2 and V3. However, improved V1V2 and V3 immunogenicity detected upon IC immunization in our studies may also be attributed to the Fc function, even though evidence for this phenomenon is still developing. Our early study examining different types of APCs treated with ICs versus un-complexed gp120 did not show the influence of Fc-enhanced uptake [59,72,73]. Rather, gp120 complexed with high-affinity CD4bs-specific mAbs was more stable and resistant to proteolytic degradation, resulting in lower gp120 antigen presentation of MHC-II-restricted CD4 T cells [52,71]. Correspondingly, lower CD4 T cell responses were elicited by vaccination with gp120/anti-CD4bs mAb ICs compared with un-complexed gp120 [46]. The complexing of gp120 with mAbs against other gp120 regions, including C-terminal C5, V3, and V2i, did not retard gp120 antigen processing and presentation [52,72], but whether these ICs effectively engage FcRs and CRs to facilitate gp120 antigen presentation to helper T cells and B cells remains unclear and requires further investigation. We should note that a major drawback of IC studies in our lab is that human IgG mAbs were used to construct the ICs tested for immunogenicity in mice, leading to the elicitation of robust anti-human IgG responses soon after a single IC vaccine administration [48]. To better incorporate the Fc contribution to the IC vaccine strategy, future studies are planned to construct IC vaccines using mAbs with a rhesus macaque Fc IgG fragment in order to evaluate their immunogenicity in this non-human primate model. In addition, IC vaccines’ improvement may be accomplished by engineering mAbs with Fc mutations or glycan modifications that enhance FcR and complement binding [15,73].

### 3.5. Lessons from Antibody Passive Transfer Studies

The immunomodulatory potential of ICs has also been alluded to in antibody passive transfer studies, which point to the “vaccinal effect” of ICs formed by the exogenous antibodies on endogenous T cell and antibody responses [74]. An early study by Haigwood et al. [75] demonstrated that the infusion of polyclonal neutralizing antibodies to SIVsmE660-infected macaques produced de novo neutralizing antibodies at a significantly accelerated pace, with levels at week 12 post-infection comparable to levels at week 32 for control macaques that received normal or no antibodies. Similarly, passive transfer of SHIV-specific IgG to SHIV-infected rhesus macaques and bnAb b12 to rhesus infants before SHIV challenge accelerated de novo neutralizing antibody production [76]. In another experiment where macaques challenged with SHIV received early administration of a single two-week course of bNAbs10-1074 and 3BNC117 in combination, all recipient animals experienced viral suppression between 7 and 25 weeks consistent with the bNAb half-life in vivo [77]. Interestingly, some animals were able to continue maintaining very low viremia for over 2 years, and virus suppression was dependent on CD8 T cells, as administration of an anti-CD8-depleting antibody resulted in immediate viral rebound. This long-lasting CD8 T cell-mediated protection was thought to be instigated by the generation of ICs between the infused bNAbs with circulating virions or antigens that engage FcRs on APCs for antigen processing and MHC-I cross-presentation, resulting in the elicitation of effective CD8 T cell response, although clear evidence for the role of ICs and their Fc engagement was not yet available [74,77]. These passive transfer studies offer an impetus to investigate and harness the effects of ICs for stimulating more efficacious antibody and T cell responses by vaccination.

## 4. Challenges in the IC Vaccine Development

In developing IC-based vaccines, a number of significant challenges must be addressed. For our IC vaccine studies, the steric and allosteric effects on Env antigenicity and immunogenicity depend greatly on the stability of ICs, requiring high-affinity mAbs and precluding low-affinity mAbs. Nonetheless, the long-term stability of these ICs must be assessed, as well as the influence of adjuvant, an essential component for vaccine formulation [78,79,80]. Chemical cross-linking to form more stable IC vaccines has been attempted [65,67], but is likely to result in the introduction of irrelevant antigenic sites sidetracking the vaccine-induced antibody responses. Our recent study also signifies the Env strain-specific effects in which a mAb induces steric or allosteric alterations only on a particular Env strain [42], necessitating the availability and screening of a relatively large mAb panel for each of the different Env strain immunogens to be developed into IC vaccines.

Finally, the potential adverse effects of ICs should be considered in the development of any IC-based vaccines, as ICs have been shown to cause immune dysregulation in chronic infectious and autoimmune diseases. IC formation and accumulation have led to the prolonged engagement of FcγR signaling and aberrant antibody responses, triggering sustained inflammatory responses in autoimmune, neoplastic, or infectious etiologies [81]. IC-mediated dysregulation may also lead to elevated type I IFN, hyperactivation of lymphocytes, or T cell exhaustion due to upregulation of immune checkpoints such as programmed cell death-1 (PD-1) and its ligand PD-L1. During chronic lymphocytic choriomeningitis virus (LCMV) infection, for instance, production of ICs has been shown to compete for available FcγR, impairing antibody-mediated clearance of opsonized target cells, cross-presentation mediated by dendritic cells, as well as antibody-mediated lymphocyte depletion by an anti-CD20 antibody used therapeutically against B cell lymphomas [82,83]. Although an IC vaccine is unlikely to elicit dysfunctional or pathogenic responses seen in virus infections, future studies to investigate the capacity of IC vaccines in triggering immune dysregulation are warranted. Such studies would also help address basic immunological questions that remain about the role of ICs and Fc fragments in antibody-mediated immunity and pathogenesis.

## 5. Conclusions

IC vaccine strategies offer promising results to improve and fine-tune the induction of antibody responses against various pathogenic microbes via Fab- and Fc-mediated immunomodulatory activities. By engaging Fc and complement receptors, ICs can bridge the innate and adaptive immune system to augment the elicitation of antibody and cellular immune responses. Fab-mediated steric and allosteric effects, on the other hand, can alter the fine specificities, affinities, and effector functions of IC-induced antibody responses. IC strategies have been tested in human vaccine trials against HBV and used for veterinary vaccines in chickens against IBDV and in pigs against PPV. IC vaccines have also been tested in animal models to direct the antibody responses toward or away from distinct epitopes on the HIV envelope glycoprotein, with varying degrees of success. While there remain significant challenges to be overcome, accumulating data from IC vaccine studies reveal important lessons that can inform the development of more effective vaccines and treatment modalities against infectious diseases.

## Data Availability

Data supporting Figure 3 are published in [42].
